# Mathematical Modeling and Numerical Analysis of Thermal Distribution in Arch Dams considering Solar Radiation Effect

**DOI:** 10.1155/2014/597393

**Published:** 2014-02-17

**Authors:** H. Mirzabozorg, M. A. Hariri-Ardebili, M. Shirkhan, S. M. Seyed-Kolbadi

**Affiliations:** ^1^Department of Civil Engineering, K. N. Toosi University of Technology, P.O. Box 15875-4416, Tehran, Iran; ^2^Department of Civil, Environmental and Architectural Engineering, University of Colorado, Boulder, CO 80309-0428, USA

## Abstract

The effect of solar radiation on thermal distribution in thin high arch dams is investigated. The differential equation governing thermal behavior of mass concrete in three-dimensional space is solved applying appropriate boundary conditions. Solar radiation is implemented considering the dam face direction relative to the sun, the slop relative to horizon, the region cloud cover, and the surrounding topography. It has been observed that solar radiation changes the surface temperature drastically and leads to nonuniform temperature distribution. Solar radiation effects should be considered in thermal transient analysis of thin arch dams.

## 1. Introduction

Performance evaluation of high arch dams is of interest in the field of structural engineering due to their complicated design methodology and catastrophic financial and loss consequences due to their failure. Thus, identification, description, and screening of the all parameters affecting the safety of arch dams are important. Dam self-weight, hydrostatic load, silt pressure, thermal loads, and seismic loads (if any) are the most important loads which are usually considered in design and analysis processes. Sources of the thermal loads in concrete dams can be categorized into the three main groups, that is, seasonal temperature and climate variation, mass concrete hydration at early ages, and solar radiation on the exposed faces. It is essential to realize the sensitivity of the thermal distribution within the thin high arch dams with respect to these parameters.

In general there are two approaches in thermal analysis of concrete dam, that is, thermal analysis of thin arch dams in which the assumption of linear temperature distribution through the dam thickness may be acceptable and solar radiation can have significant effect on structural responses of the whole dam body and thermal analysis of the relatively thick sections (i.e., arch-gravity, gravity, and RCC dams) in which nonlinear temperature distribution through the body thickness is expected. In the second case, concrete temperature near the dam face (especially downstream face) changes quickly with respect to the air and water temperature while the temperature inside the body has no meaningful variation. Thus, the solar radiation may affect only the temperature of the exterior areas with no considerable effect on the interior sections [1].

Agullo et al. [2] measured the temperature of each node in numerical model of a concrete dam using explicit finite difference method. They used energy conversion factor for radiation modeling and Newton's law for simulation of the thermal convection. Meyer and Mouvet [3] investigated thermal behavior of a concrete gravity dam in Switzerland utilizing finite element method. They found that the air temperature fluctuation is not a lonely good criterion for estimation of concrete dam's temperature. Solar radiation increases the whole dam's temperature of about 4°C and the upstream water could regulate the temperature fluctuation amplitude. Castellanos and Marin [4] studied the effect of various parameters on thermal behavior of the concrete arch and gravity dams using a three-dimensional (3D) finite element model. B. Zhu [5] proposed an analytical formula as boundary condition of heat transfer analysis in concrete dams in order to estimate the water temperature through the deep reservoirs. Sheibany and Ghaemian [6] developed a detailed 3D finite element model of an arch dam for estimation of thermal stresses. They found that probable cracks occur in a very narrow region of the downstream face which is mainly due to thermal loads in comparison with self-weight and hydrostatic loads. Ardito et al. [7] studied diagnostic damage analysis of a concrete arch-gravity dam based on seasonal hydrostatic loading considering thermal effects. Jin et al. [8] investigated a practical procedure for predicting nonuniform temperature on the exposed faces of the arch dams due to variation in solar radiation and shading effects. They found that the temperature distribution on the upstream face is mainly due to the reservoir temperature condition, while on the downstream face the temperature distribution is mainly influenced by the solar radiation. They showed that the nonuniform temperature has a significant effect on the surface thermal stresses and crack propagation. K. H. Zheng and B. Y. Zheng [9] studied coupled thermal-creep-stress problem for high arch dams using finite element method. Yang et al. [10] developed a finite element program for thermal analysis of mass concrete embedded with double-layer staggered heterogeneous cooling water pipes based on the equivalent equation of heat conduction including the effect of cooling water pipes and hydration heat of concrete. Comparing calculated results with actual measured data from a monolith of an arch dam in China, their numerical model was proven to be effective in simulating accurate temperature variations of mass concrete. Yangbo et al. [11] proposed two fast algorithms to overcome the huge and time-consuming finite element analysis on temperature control and thermal stress fields of concrete dams' construction. One was overall planning algorithm and the other was incomplete Cholesky conjugate gradient algorithm. By applying these two to the temperature forecasts, it was shown that the calculated results are accurate. Fujun et al. [12] studied the cracking reasons of concrete overflow dam of Hadashan Hydro Project by developing a finite element method to simulate the temperature distribution and thermal stress analysis during the construction period. Results have shown that the crack of the concrete overflow dam is a temperature crack due to combined action of the internal thermal gradient and the external restraints.

In the present paper, impact of the solar radiation on thermal distribution in a high thin arch dam is investigated by solving the 3D differential equation governing the phenomenon within the mass concrete and finally the results are compared with those obtained from recorded data from the dam site.

## 2. Mathematical Model for Heat Transfer

### 2.1. Thermal Conduction

Conduction (or heat conduction) is a mode of transfer of energy within and between bodies of matter due to a temperature gradient. Governing equation of thermal conduction in continuous environments can be achieved according to the conservation principle of thermal energy on constant arbitrary volume, *V*, surrounded by closed surface, *S*. Implementation of heat transfer relation for a system including anisotropic material is as follows:

(1) $$\begin{array}{c} \rho C\frac{\partial T}{\partial t}=\mathrm{div}\left(\vec{k}\;\text{grad}T\right)+Q, \end{array}$$

where *ρ* is density, *C* is specific heat, *T* is temperature of medium, *t* is time, $\vec{k}$ is a tensor representing thermal conduction coefficient, and *Q* is internal heat generated per volume.

### 2.2. Thermal Convection

The amount of heat transferred by convection is governed by Newton's cooling law as follows:

(2) $$\begin{array}{c} q_{c}=-h_{c}\left(T_{a}-T_{\text{sb}}\right), \end{array}$$

where *h*_*c*_ is convective coefficient, *T*_sb_ is temperature of surface boundaries, and *T*_*a*_ is ambient temperature. Convective coefficient is a function of fluid velocity, *V*_*f*_, and its properties such as type of flow, viscosity, and surface roughness. There are many simplified formulas for calculating this coefficient such as the one proposed by Duffie and Beckman [13]:

(3) $$\begin{array}{c} h_{c}=3.8V_{f}+5.7. \end{array}$$

### 2.3. Thermal Radiation

Thermal radiation is an electromagnetic radiation generated by the thermal motion of charged particles in matter. All matter with a temperature greater than absolute zero emits thermal radiation. In the present analysis, radiation involved at the issue is in two forms, that is, radiation energy absorbed by the surfaces and electromagnetic energy released from the surface which is known as thermal radiation. The total amount of the absorbed energy is expressed as [14]

(4) $$\begin{array}{c} q_{a}=aI_{t}, \end{array}$$

where *a* is solar absorptivity of surface and *I*_*t*_ is total solar energy on surface. Thermal radiation caused by the temperature difference between surface and its environment can be achieved by Stefan-Boltzmann law:

(5) $$\begin{array}{c} q_{r}=-eC_{s}\left(T_{a}^{4}-T^{4}\right), \end{array}$$

where *e* is emissivity of surface, that is, the ratio of heat released by intended surface to heat released by a black body with the same temperature, and *C*_*s*_ = 5.669 × 10^−8^ Wm^−2^K^−4^ is Stefan-Boltzmann constant. It should be noted that the wasted heat between surface and air due to radiation is not significant. Thus, (5) can be rewritten as a pseudolinear equation as follows:

(6) $$\begin{array}{c} q_{r}=-h_{r}\left(T_{a}-T\right), \end{array}$$

where *h*_*r*_ is defined as

(7) $$\begin{array}{c} h_{r}=eC_{s}\left(T_{a}^{2}+T^{2}\right)\left(T_{a}+T\right). \end{array}$$

Therefore, the radiation effects can be considered by adding *h*_*r*_ to *h*_*c*_ and obtaining new convective coefficient.

## 3. Boundary Condition for Heat Transfer Equation

**Figure 1:**
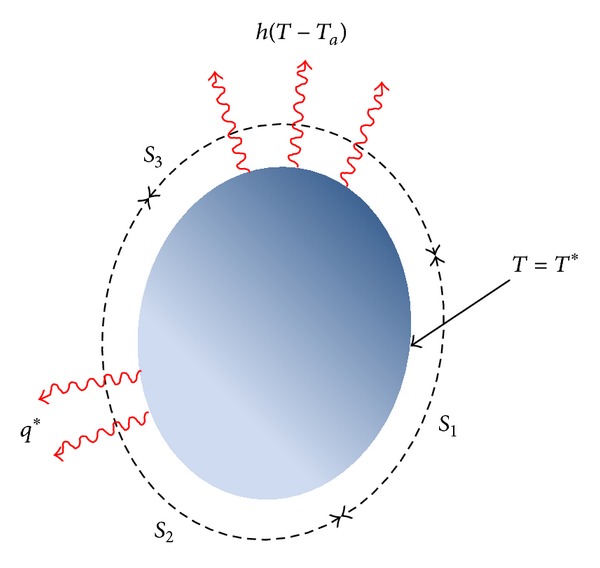
Various types of boundary conditions in 3D heat transfer [15].

**Figure 2:**
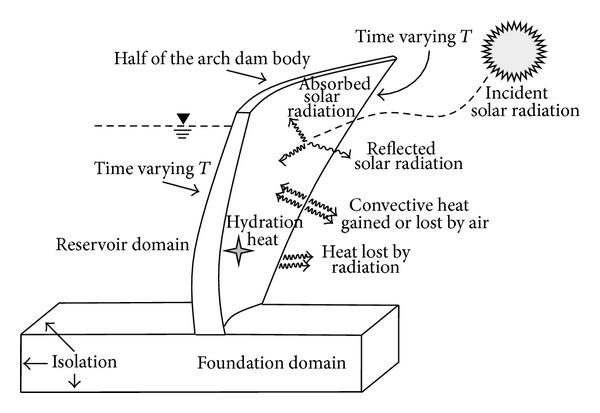
Heat transfer mechanism for arch dams in 3D space.

The numerical solution of differential equation requires information as known quantities applied to the systems in the form of boundary conditions. Boundary conditions of three-dimensional conduction with convection are shown in Figure 1 schematically including imposed constant temperature on the boundary *S*_1_, imposed heat flux on the boundary *S*_2_, and the edge convection condition on the boundary *S*_3_. Also, the environmental conditions in a typical arch dam that are important in heat transfer analysis and its detailed methodology are shown in Figure 2.

### 3.1. Air Temperature

Estimation of the dam site's air temperature usually depends on the data obtained from the nearest weather station. In the current study, the following data were collected from weather stations:

- daily mean temperature, *T*_*i*_,
- daily maximum (*T*_max⁡_)_*i*_ and daily minimum temperatures (*T*_min⁡_)_*i*_,
- the maximum monthly mean temperature, *T*_max⁡_,
- the minimum monthly mean temperature, *T*_min⁡_,
- annual mean temperature, *T*_mean_, and
- the highest and lowest recorded temperatures at the site.

**Figure 3:**
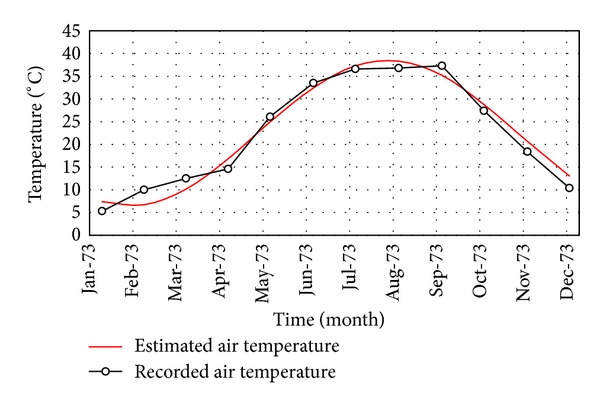
Comparison of the air temperature between the recorded data and the estimated ones.

If there is no information about the air temperature at the dam site, it is possible to use the sinusoidal function with the acceptable accuracy as

(8) $$\begin{array}{c} T_{a}\left(t\right)=\overset{-}{A}\sin\left(\frac{2\pi \left(t-\xi \right)}{P}\right)+\overset{-}{B}, \end{array}$$

where $\overset{-}{A}$ is the amplitude, $\overset{-}{B}$ is the annual mean temperature, *t* is time in day, *ξ* is time in day in which $T_{a}\left(\xi \right)=\overset{-}{B}$, and *P* is period of sine function, which here is 365 days. In this function $\overset{-}{A}$ can be obtained as

(9) $$\begin{array}{c} \overset{-}{A}=\frac{1}{2}\left(\left|T_{\max}-T_{\text{mean}}\right|+\left|T_{\min}-T_{\text{mean}}\right|\right), \end{array}$$

in which,

(10) $$\begin{array}{c} T_{\max}=\frac{\sum_{i=1}^{n}{\left(T_{\max}\right)}_{i}}{n},\\ T_{\min}=\frac{\sum_{i=1}^{n}{\left(T_{\min}\right)}_{i}}{n},\\ T_{\text{mean}}=\frac{\sum_{i=1}^{P}T_{i}}{P}, \end{array}$$

where *n* is the number of days at month.  Figure 3 compares the recorded and estimated air temperatures for one of the stations, in the current research, as a sample.

### 3.2. Reservoir Temperature

**Figure 4:**
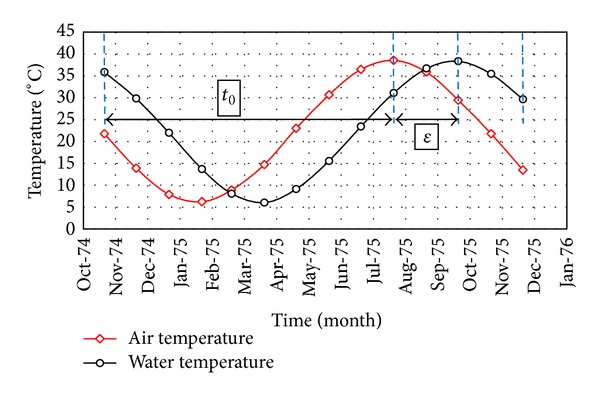
Physical concept of the parameters *ε* and *t*_0_.

Distribution of the temperature within the dam body is strongly affected by the reservoir water temperature. The best source for this information is the collected statistical data from the similar reservoirs. This type of information is collected by USBR and reported in engineering supplement number 34 [16]. The proposed method by Bofang [5] is another way for estimation of the reservoir temperature which is used in the present study (11). Bofang showed that the proposed method is in good agreement with the recitation values from China reservoirs and can be used for other reservoirs with small changes in the parameters:

(11) $$\begin{array}{c} T\left(y,t\right)=T_{m}\left(y\right)+A\left(y\right)\cos\omega \left(t-t_{0}-\varepsilon \right), \end{array}$$

where *y* is reservoir depth, *t* is time in day, *T*(*y*, *t*) is the reservoir temperature at time *t* and depth *y*, *T*_*m*_(*y*) is the mean annual temperature of the reservoir, *A*(*y*) is the amplitude of the annual variations in the reservoir temperature, *ω* is the frequency of temperature variations, and the parameters *ε* and *t*_0_ are illustrated in Figure 4.

The mean annual temperature of the reservoir, *T*_*m*_(*y*), can be obtained as follows [5]:

(12) $$\begin{array}{c} T_{m}\left(y\right)=c+\left(T_{s}-c\right)e^{-\alpha y},\\ c=\frac{\left(T_{b}-gT_{s}\right)}{\left(1-g\right)},\\ g=e^{-0.04H}, \end{array}$$

where *T*_*s*_ is the mean annual temperature of the reservoir at the surface, *T*_*b*_ is the mean annual temperature of the reservoir at the bottom, *H* is the reservoir depth, and the parameter *α* is 0.04. The detailed formulations on how *T*_*s*_ and *T*_*b*_ are calculated can be found in Bofang [5]. Moreover, the amplitude of the annual variations in the reservoir temperature, *A*(*y*), is

(13) $$\begin{array}{c} A\left(y\right)=A_{0}e^{-\beta_{0}y},\\ A_{0}=\frac{T_{\max}-T_{\min}}{2}. \end{array}$$

Parameter *β*_0_ is a function of reservoir height and is calculated as:

(14) $$\begin{array}{c} \beta_{0}=-0.058545+7.2727\times 10^{-4}\times H. \end{array}$$

The minimum allowed value for *β*_0_ is 0.018. Finally the parameter *ε* may be calculated by the following equation:

(15) $$\begin{array}{c} \varepsilon =d-fe^{-\gamma y}, \end{array}$$

where the parameters *γ*, *d*, and *f* are 0.085, 2.15, and 1.30, respectively.

### 3.3. Solar Radiation Effect

Solar radiation, according its nature, leads to increasing of temperature at the exposed surfaces of the dam body and is a function of the slope, direction, and latitude of the surface with respect to sun. The navigation for all parts of the gravity dams is similar and only one value for each side of the dam body would be required, while this value should be calculated for several locations of an arch dam with respect to its shape.

**Figure 5:**
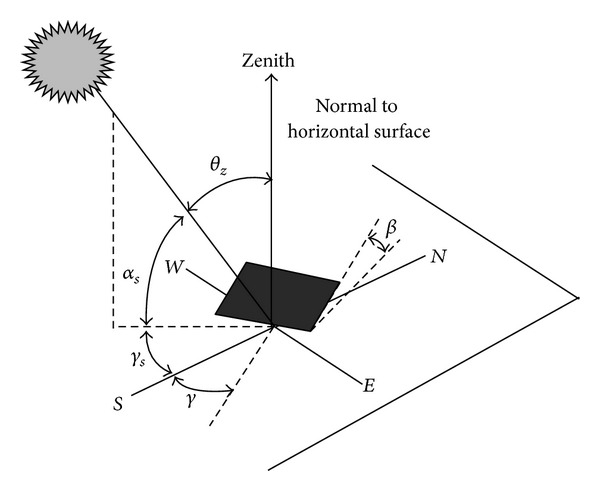
3D view of the angles due to solar radiation.

The amount of the solar radiation received by an arch dam depends on a series of periodic seasonal changes. This variation is a function of different factors such as the height of the dam site above the sea level, surface directions relative to sun, surface slope relative to horizon, the region cloud cover, surrounding topography of the dam site, and the time of the year. According to the above definitions, the total amount of radiation absorbed by the surface can be calculated from (4). In order to have a clear vision of the total solar energy received on an inclined surface, *I*_*t*_, Table 1 and Figure 5 summarize various required angles in the solar radiation problem. Consequently, total solar energy received on an inclined surface can be calculated as [16]

(16) $$\begin{array}{c} I_{t}=R_{b}\left(D-D_{d}\right)+D_{d}\left(\frac{1+\cos\beta }{2}\right)+\rho_{d}D\left(\frac{1-\cos\beta }{2}\right), \end{array}$$

where *D* is total days that solar is radiated on horizontal surface on the ground, *D*_*d*_ is daily diffused solar radiation on horizontal surface on the ground, and *ρ*_*d*_ is the diffuse reflectance coefficient of the surrounding surface, generally ground surface, and reservoir in issue of arch dams and is given in Table 2 for various surrounding conditions.

Parameter *R*_*b*_ is a geometric factor and is defined as the ratio of direct radiation on the inclined surface to the horizontal surface and is derived as

(17) Rb=[(cos⁡βsinδsinϕ)(ωss−ωsr)(π180)−(sinδcos⁡ϕsinβcos⁡δ)(ωss−ωsr)(π180)+(cos⁡ϕcos⁡δcos⁡β)(sinωss−sinωsr)+(cos⁡δcos⁡γsinϕsinβ)(sinωss−sinωsr)−(cos⁡δsinβsinγ)(cos⁡⁡ωss−cos⁡⁡ωsr)] ×[2cos⁡⁡ϕcos⁡⁡δsin⁡ωs+(π180)ωssinδsinϕ]−1,

where *ω*_*ss*_ and *ω*_*sr*_ are calculated as follows:

(18) ωss=(−min⁡{ωs,cos⁡−1((AB+A2−B2+1)A2+1)},γ≥0−min⁡{ωs,cos⁡−1((AB−A2−B2+1)A2+1)},γ≥0),ωsr=(min⁡{ωs,cos⁡−1((AB−A2−B2+1)A2+1)},γ≥0min⁡{ωs,cos⁡−1((AB+A2−B2+1)A2+1)},γ≥0).

Values of *A*, *B*, and *ω*_*s*_ are calculated using the following relations:

(19) $$\begin{array}{c} A=\frac{\cos\phi }{\sin\gamma \tan\beta }+\frac{\sin\phi }{\tan\gamma },\\ B=\tan\delta \left(\frac{\cos\phi }{\tan\gamma }-\frac{\sin\phi }{\sin\gamma \tan\beta }\right),\\ \omega_{s}=\cos^{-1}\left(-\tan\phi \tan\delta \right). \end{array}$$

The total days that solar is radiated, *D*, can be considered as the sum of direct and indirect radiations. Because of different orientation and slope of various locations on the exposed surfaces of arch dams, it is necessary to evaluate the direct and indirect solar radiation. Thus, the Angstrom equation ((20)—2nd part) is used to get the amount of in place sunny hours. Consider

(20) $$\begin{array}{c} D=D_{d}+D_{b}\;\text{or}\;\frac{D}{D_{0}}=a+b\left(\frac{n^{\prime }}{N}\right), \end{array}$$

where *a* and *b* are experimental constants depending on geographical location of the dam. Their values for Dez Dam, as case study in the present paper, are 0.2515 and 0.446, respectively. Continuously, parameter *n*′ is mean monthly sunny hour in station, *N* is the sunny day length for each month that can be calculated from (21), and *D*_0_ is monthly extraterrestrial radiation on horizontal surface which can be calculated using (22):

(21) $$\begin{array}{c} N=\frac{2}{15}\omega_{s}, \end{array}$$

(22) D0=24×3600πGsc(1+0.033cos⁡360n365) ×(cos⁡ϕcos⁡δsinωs+2πωs360sinϕsinδ),

where *G*_sc_ is solar constant and is equal to 1353 Wm^−2^ and *n* is number of the day in AD year. Parameter *D*_0_ is calculated using (22) on a daily basis while if the mean monthly of *D*_0_ is intended, it should be extracted from Table 3. Knowing the values of *D* and *D*_0_, *D*_*d*_ can be calculated using (23) and finally we have the total radiation on the inclined surface:

(23) Dd=D[0.775+0.00653(ωs−90)−(0.505+0.00455(ωs−90))×cos⁡(115DD0−103)].

## 4. Finite Element Implementation of Heat Transfer

In the present study, finite element technique is used for numerical implementation of the heat transfer problem within the dam body. The mathematical description of heat transfer relation for a system including anisotropic material in 3D space was explained in (1). Using the appropriate boundary conditions, as discussed in previous sections, it is possible to define the general matrices for heat transfer as [15]

(24) $$\begin{array}{c} \left[C^{\left(e\right)}\right]\left\{{\dot{T}}^{\left(e\right)}\right\}+\left[K^{\left(e\right)}\right]\left\{T^{\left(e\right)}\right\}=\left\{f_{Q}^{\left(e\right)}\right\}+\left\{f_{q}^{\left(e\right)}\right\}, \end{array}$$

where {*T* ^(*e*)^} is temperature vector, [*C* ^(*e*)^] is element conspecific heat matrix, [*K* ^(*e*)^] is element conductivity matrix, {*f*_*Q*_^(*e*)^} is element heat generation vector, and {*f*_*q*_^(*e*)^} is element convection surface heat flow vector and all are defined as follows:

(25) [K(e)]=∭V[kx∂[N]T∂x∂[N]∂x+ky∂[N]T∂y∂[N]∂y+kz∂[N]T∂z∂[N]∂z]dV,[C(e)]=∭Vρc[N][N]TdV,{fQ(e)}=∭VQ[N]TdV,{fq(e)}=−∯A(qxnx+qyny+qznz)[N]TdA,

where *n*_*x*_, *n*_*y*_, and *n*_*z*_ are unit normal vectors on the considered surface and *q*_*x*_, *q*_*y*_, and *q*_*z*_ are defined as

(26) $$\begin{array}{c} q_{x}=k_{x}\frac{\partial T}{\partial x}N_{i},\\ q_{y}=k_{y}\frac{\partial T}{\partial y}N_{i},\\ q_{z}=k_{z}\frac{\partial T}{\partial z}N_{i}, \end{array}$$

where *k*_*x*_, *k*_*y*_, and *k*_*z*_ are conduction coefficients of mass concrete in *x*, *y*, and *z* global directions, respectively, and, finally, *N*_*i*_ is the shape function for the *i*th node in the considered thermal element.

## 5. Numerical Model of Arch Dam 

### 5.1. Arch Dam Description

**Figure 6:**
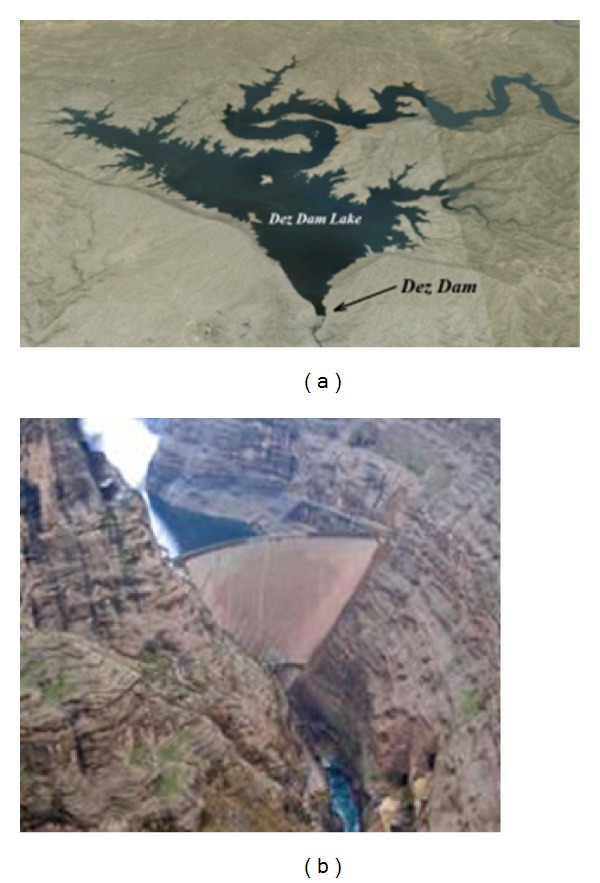
(a) Satellite view of Dez Dam and the lake [17]; (b) general view of Dez Dam, river, and the reservoir [18].

Dez Dam is located at 20 km NE of Andimeshk, Khuzestan, Iran (Figure 6(a)). This dam is considered as a thin double curvature arch dam. The dam height is 203 m from the foundation and 190 m from the river bottom. Its thickness is 4.5 m at the crest and 21 m at the foundation base level of the crown cantilever [18]. The dam crest length is 212 m and its crest level is 354 asl (above sea level). The maximum operation level of the dam was designed at an altitude of 350 asl. In order to optimize the dam and its hydropower operation, it has been increased to the level of 352 asl [18].

A concrete Pulvino was provided between the concrete body and the foundation rock for suitable load transfer from the dam body to its abutments. There is an overall joint between the dam body blocks and the Pulvino called peripheral joint.  Figure 6(b) shows the downstream view of the dam and its surrounding environment.

### 5.2. Thermal Finite Element Model

**Figure 7:**
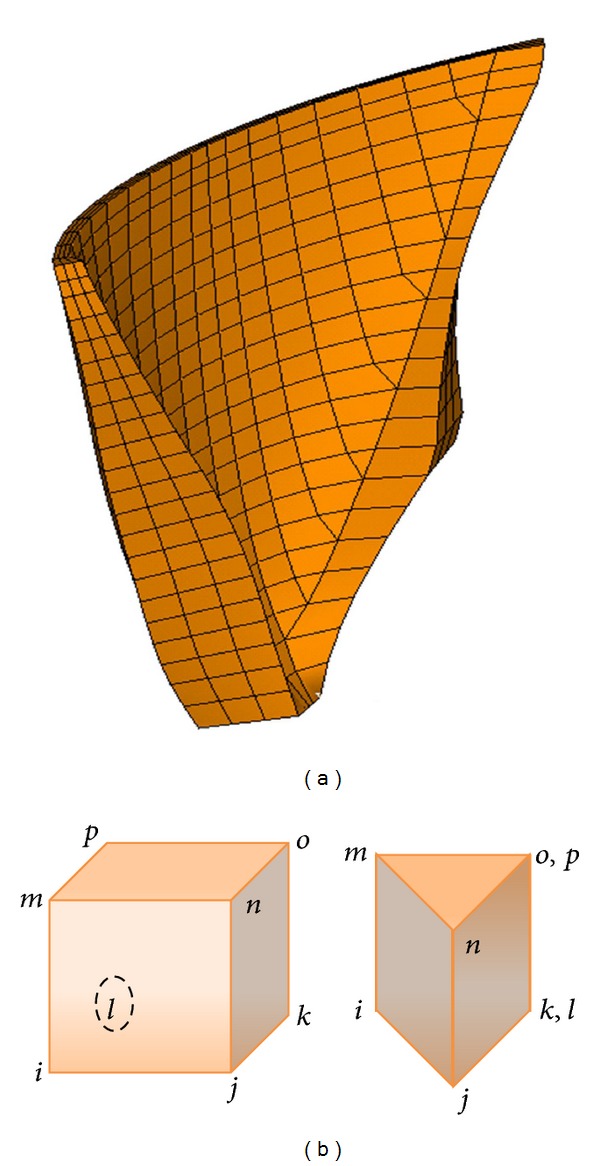
(a) Finite element model of Dez Dam for thermal analysis; (b) cubed and prism solid thermal elements.

A finite element model of the dam body was provided for thermal transient analysis. The provided model includes 1748 isoparametric linear elements in three layers through the thickness connected through 2120 nodes (Figure 7(a)). Mostly, eight-node solid thermal elements with one thermal degree of freedom were used for modeling while some prism elements were used in vicinity of Pulvino (Figure 7(b)).

### 5.3. Applied Initial Temperature

**Figure 8:**
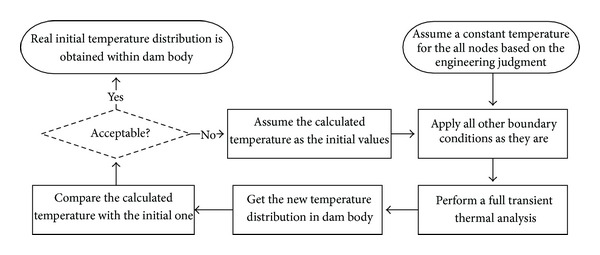
Proposed algorithm for calculation of the real initial temperature distribution.

In order to perform a valid thermal analysis, it is required to know the initial temperature (reference temperature) at the system. Considering that Dez Dam is about 52 years old with limited instrumentation within the dam body, there is no reliable information about initial temperature distribution within the body [18]. So a heuristic method is used in the current study for determination of the initial temperature. In the first step, a constant temperature of 23°C is applied to the all nodes which is defined by tracking the similar dams and the engineering judgment. Then, thermal transient analysis is performed for one year considering the imposed boundary conditions. Results of the thermal distribution on the nodes at the end of this step are applied to the model again as the new initial conditions for the next step and this loop is repeated for several years until approaching the stable responses. The results of the thermal distribution at the end of the last step can be interpreted as real initial temperature of the model after several years.  Figure 8 shows the proposed algorithm for calculating the initial temperature distribution on the dam body.

**Figure 9:**
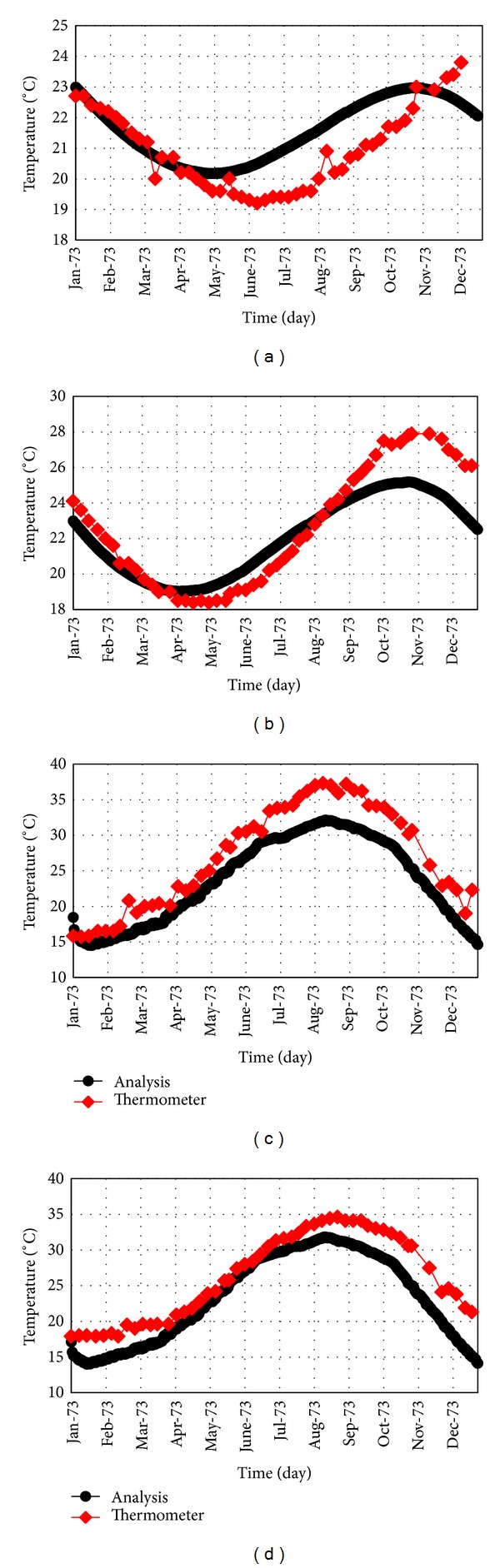
Comparison of the results obtained from thermal analysis and thermometers; (a) middle node at 280 m asl; (b) middle node at 316.5 m asl; (c) downstream node at 280 m asl; (d) downstream node at 316.5 m asl.

Figure 9 compares the results obtained from numerical analysis with the data collected from thermometers installed in block number 9 corresponding to year 1973. Results indicated that the responses from thermal analysis are in good compliance with the actual values. It should be mentioned that there is no middle point in numerical model and the results for this point are the average of two side nodes and some differences between the actual and theoretical data are expected.

### 5.4. Determination of Meteorological Parameters

#### 5.4.1. Air Temperature

**Figure 10:**
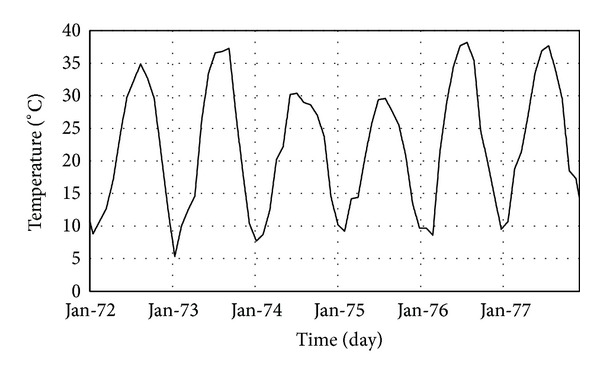
Variation of the air temperature recorded in Dez Dam site.

As mentioned before both the recorded data and proposed sinusoidal function in (8) could be used for determination of the air temperature at the dam site. Considering that the nearest meteorological station to the dam site is station number 21-144, so these results are used as reference. Air temperature is recorded monthly in this station, so it is required to use the linear interpolation in order to extract daily air temperature.  Figure 10 shows the sample of air temperature variation for the period of six years in this station.

#### 5.4.2. Reservoir Water Temperature

**Figure 11:**
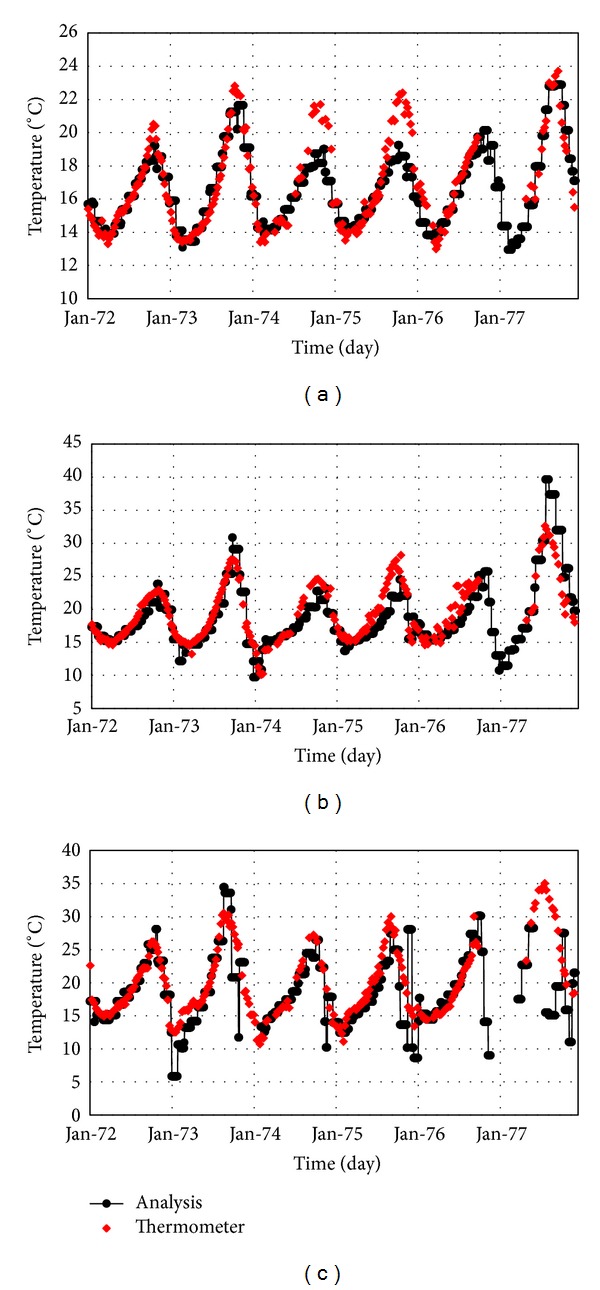
Comparison of the results obtained from thermal analysis and thermometers at upstream face of block number 9: (a) level 280 m asl; (b) level 316.5 m asl; (c) level 328 m asl.

The empirical-analytical Bofang's relations are used to calculate the temperature of the reservoir water [5]. Using (11)–(15) and considering *d* = 2.15 and *f* = 1.30, it is possible to simulate the water temperature at different elevations. A sample of computational results in comparison with values recorded by the water temperature thermometers *T*_*w*_ at the upstream face in block number 9 is shown in Figure 11. According to these figures, the calculated values show good agreement with the real values. The differences between analysis and thermometer data for level 328 m asl is due to decreasing reservoir water in some months to lower than this level and so this part of the dam is in expose of the air temperature instead of the water.

#### 5.4.3. Effect of Solar Radiation

**Figure 12:**
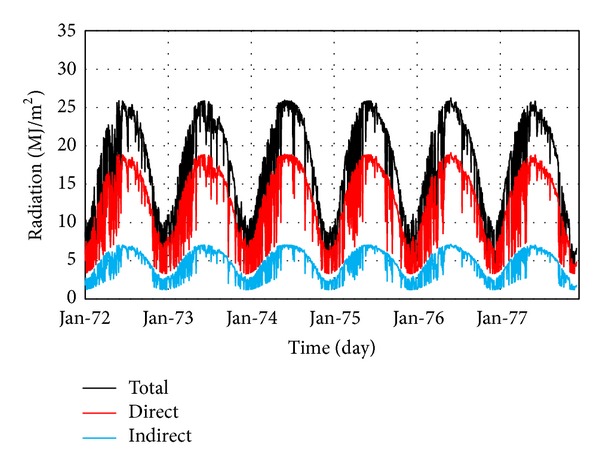
Estimated values for the direct, indirect, and total radiation.

In order to implement the solar radiation, the direct and indirect values of daily radiation on horizontal plane should be determined. Due to the lack of the required instruments for measuring radiation near the dam site, its amount is determined according to the recorded daily sunshine hours at the Dez station for several years and calculated using (12)–(16). Direct, indirect, and total radiations calculated for the six years are shown in Figure 12.

**Figure 13:**
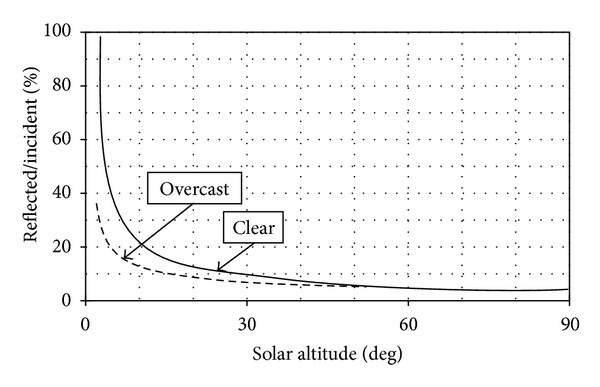
Ratio of the total reflected radiation to the total radiation at the water surface according to sun height and smooth/cloudy sky.

The reflection coefficient is assumed to be 0.2 for the region surrounding the site and 0.3 for water surface. Estimated coefficient of the surface reflectance values and variation of reflection coefficient from water surface are shown in Table 3 and Figure 13, respectively. Also the absorption coefficient for the body concrete is considered to be 0.7.

#### 5.4.4. Reservoir Water Level Changes

**Figure 14:**
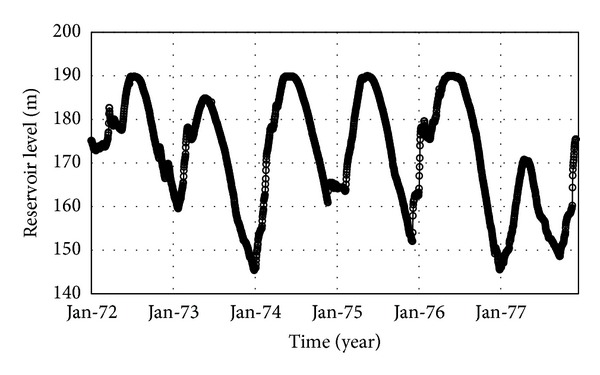
Variation of the reservoir level in Dez Dam site.

In order to properly investigate the environmental temperature effects on the thermal distribution of the dam body, the reservoir water level was tracked carefully corresponding to reservoir operation regime. To do so, the upper part of the upstream face, which is not in contact with water, is detected based on reservoir daily water level. At each step of thermal transient analysis, the lower part of the upstream face, immersed in water, is affected only by reservoir temperature while the part out of the water is affected by the air temperature and solar radiation.  Figure 14 shows the variation in water level for the period of six years.

**Figure 15:**
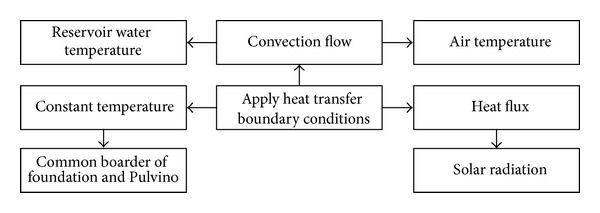
Applied thermal boundary conditions to the finite element model.

In the thermal analysis process, the first step is to determine daily thermal parameters such as air temperature surrounding the dam, water reservoir temperature, water level variations, and radiation energy. The second step is implementation of these values in the thermal transient analysis. In the current study, thermal transient analysis is performed for a time period of six years and the air and water temperatures are applied to the model as a convective flow while the solar radiation is applied to respective nodes as heat flow. Also, the foundation temperature was considered to be constant. The overview of applied thermal boundary conditions to the finite element model is shown in Figure 15.

## 6. Numerical Results versus Recorded Data 

### 6.1. Thermal Calibration of Model

**Figure 16:**
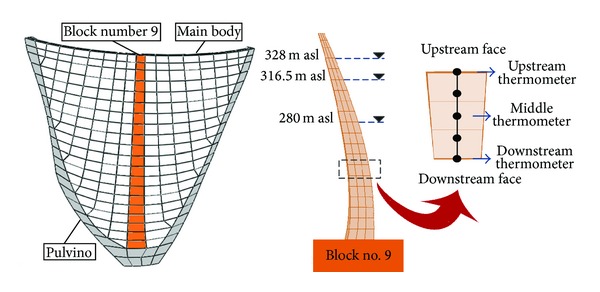
Location of the reference block and thermometers position.

**Figure 17:**
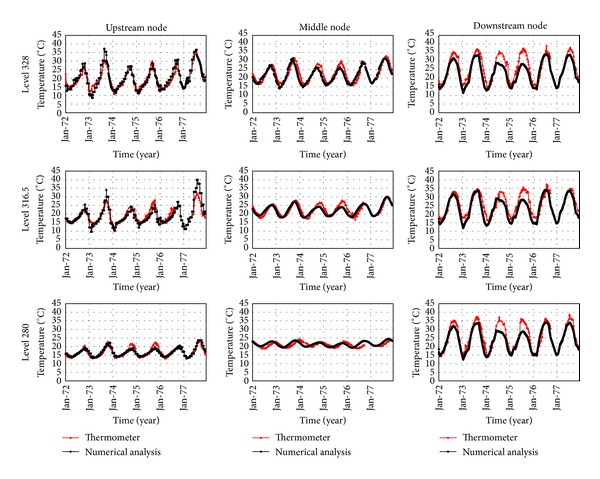
Comparison of the results from thermal calibration analysis with thermometers data.

The model calibration was performed comparing the calculated data with the actual values recorded by thermometers at block number 9, which is the only instrumented block in the Dez Dam. The calibration process was conducted for six years, 1972 to 1977, because only the data for these years were available. To do so, the initial thermal condition was determined for January 1972 and thermal transient analysis was performed using a daily time step. In other words, there were 2190 load steps in thermal transient analysis.  Figure 16 shows the location of block number 9 in the dam and also the position of the thermometers at various levels and sections.  Table 4 represents the initial and final values of important parameters which the calibration of the finite element model is based on. All these parameters were obtained after almost wide range sensitivity analysis on the responses. Finally, Figure 17 shows the comparison of the results of calibrated model at various levels and sections with those recorded by thermometers for considered time period at reference block. Results confirmed that the provided thermal model considering the solar radiation effects is able to model the real conditions and, therefore, can be used for predicting the thermal behavior of the dam body.

### 6.2. Thermal Analysis considering Solar Radiation Effects

**Figure 18:**
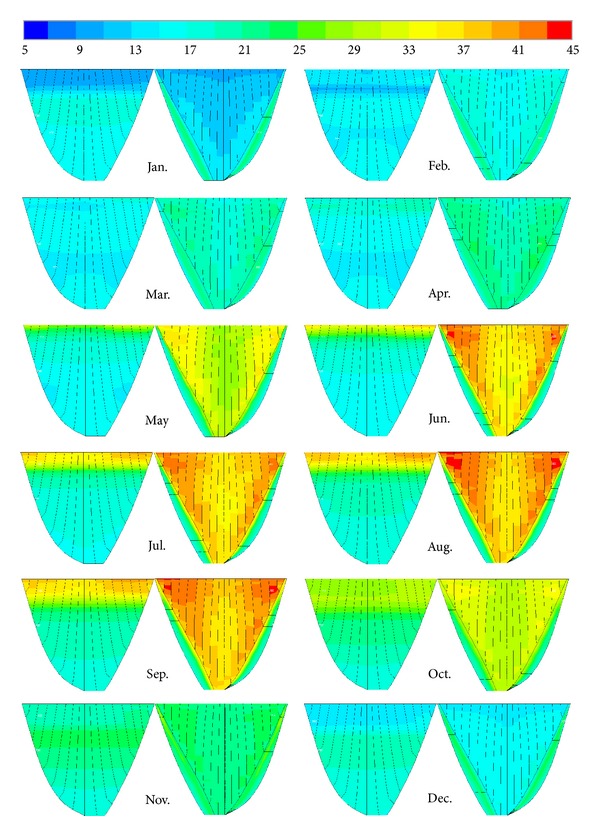
Temperature distribution on the upstream and downstream surfaces of the dam for the year 1973 considering solar radiation effects (°C).

According to Figure 18, solar radiation has an undeniable role at creation of the nonuniform temperature distribution on the dam surfaces. It means that the temperature differences between some coincident nodes highly affected by solar radiation are about 6°C to 10°C for the most hot and cold month, respectively. Also, the reservoir water fluctuation is effective in temperature controlling of the parts under water level on the upstream face. In the arch dams, their own especial geometry leads to heat concentration at some areas even at the same level. Due to location of Dez Dam which is in northern of the earth and considering that the axis of the dam has a slight angle (about 6°) to the geographic south, the sun which is located in the southern glows to downstream face and leads to more heat concentration near the abutments and middle elevations.

The effect of direct solar radiation is almost negligible on the upstream face because it is fully behind the sun. This distribution is affected by indirect radiation reflected from the environmental surfaces, which is almost insignificant. The contours depicted in Figure 18 also show the effects of reservoir water level and temperature on thermal distribution of the upstream face. Temperature variation is not considerable in the lower part of the dam, especially along the same level, because the concrete in these regions is affected mostly by the reservoir temperature instead of the air temperature and solar radiation.

### 6.3. Thermal Analysis Neglecting Solar Radiation Effects

**Figure 19:**
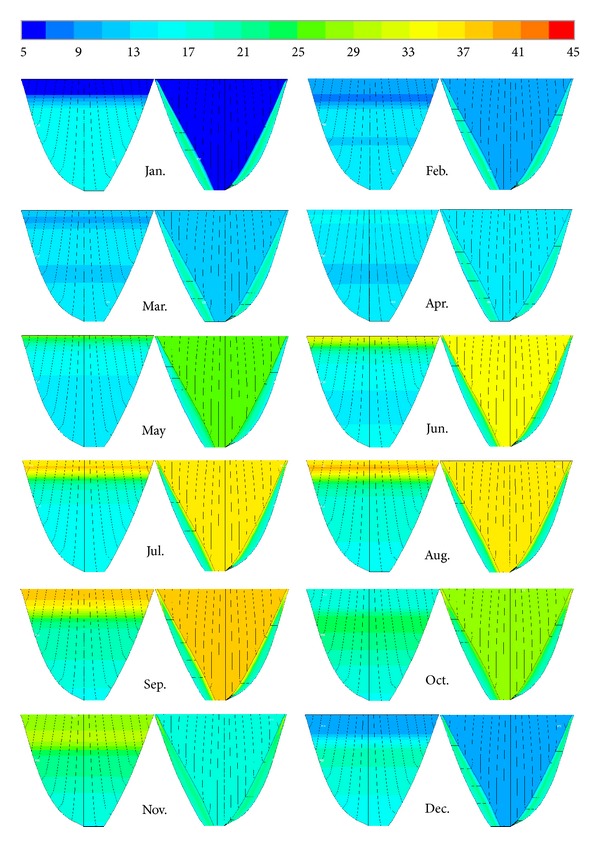
Temperature distribution on the upstream and downstream surfaces of the dam for the year 1973 neglecting solar radiation effects (°C).

Figure 19 shows the temperature distribution in the dam body neglecting the solar radiation effect. It can be clearly seen that the solar radiation had a lot of effects on temperature distribution. Neglecting solar radiation effects at the downstream face leads to completely uniform temperature distribution because only the air temperature acts as the thermal boundary condition. On the other hand the same trend is shown on the upstream face in which temperature distribution changes only by changing reservoir levels and there is no nonuniform temperature at the nodes located at the same level.

## 7. Conclusion

Thermal transient analysis of a double curvature high thin arch dam was studied in the present paper considering solar radiation effect. Air temperature, water temperature in various levels of the reservoir, and also its fluctuations were considered in the present study. Solar radiation effects were implemented in finite element model considering factors such as the height of the dam site above sea level, surface directions relative to the sun, surface slope relative to horizon, the region cloud cover, surrounding topography of the site, and the time of the year. Also the effects of direct and indirect solar energy absorption were investigated.

It is obvious that the temperature distribution on the upstream in two cases is equal except where the water level is below the thermometer's level. In this case, the temperature distribution on the upstream surface is changed due to the effect of solar radiation. According to the results of thermal analysis, the following can be concluded.

- The used boundary conditions provide realistic responses from the numerical models according to the results obtained from calibration of the finite element model.
- The estimated reservoir water temperature from empirical-analytical Bofang's relation shows a good agreement with recorded values.
- Comparison of temperature distribution on the upstream face represents that the parts lower the water level are mostly affected by reservoir temperature and its fluctuations and have uniform temperature distribution along the same levels, while the part above the water level is affected mainly by solar radiation and air temperature which leads to nonuniform thermal distribution along the various levels.
- Comparing the temperature distribution on the downstream face shows that it is affected mostly by solar radiation and air temperature, which leads to nonuniform distribution of temperature along different levels. Due to double curvature shape of the considered dam, the areas in vicinity of the abutments have more variation in temperature than the central parts.
- Radiation effect reveals up to 10°C temperature difference between coincident nodes especially on the downstream face when the effects of sunlight are considered.
- Comparison of temperature distribution which resulted from finite element method and recorded data through the thickness of the dam at different levels shows that the analytical results can estimate results of thin arch dams with good accuracy.

## Figures and Tables

**Table 1:** Summary of the important angles in solar radiation problems.

Symbol	Name	Definition
*φ*	Latitude	The angular position of the given surface to the equator
*δ*	Declination	The angular position of the sun at the solar noon to the equator
*β*	Slop	The angle between the given surface to the horizon
*γ*	Surface azimuth angle	Illustration diversion of surface normal vector on the horizontal plane to the south latitude
*ω*	Hour angle	The angular distance of the sun's east and west to origin meridian due to earth rotation in rate of 15 degrees per hour around its axis
*θ*	Angle of incident	The angle between the direct radiation on the surface and its normal vector
*θ*_*z*_	Zenith angle	The angle between the vertical line and the line along the sun
*α*_*s*_	Solar height angle	The angle between sun and horizon
*γ*_*s*_	Solar azimuth angle	The angular distance between the geographic southern and the sun illustration on horizon

**Table 2:** Estimated value of the surface reflection coefficient.

Ground condition	*ρ*
Meadows and fields	0.14
Leaf and needle frost	0.17
Dark, extended mixed frost	0.045
Health	0.1
Flat ground grass covered	0.2–0.33
Flat ground, rock	0.12–0.15
Sand	0.18
Vegetation early summer, leaves with high water content	0.19
Vegetation late summer, leaves with low water content	0.29
Fresh snow	0.83
Old snow	0.42–0.70

**Table 3:** Recommended values of *n* for different months.

Month	*n* value for *i*th day of month	For the average day of the month	
		*n*th day of month	*n*th day of year
January	*i*	17	17
February	31 + *i*	16	47
March	59 + *i*	16	75
April	90 + *i*	15	105
May	120 + *i*	15	135
June	151 + *i*	11	162
July	181 + *i*	17	198
August	212 + *i*	16	228
September	243 + *i*	15	258
October	273 + *i*	15	288
November	304 + *i*	14	318
December	334 + *i*	10	344

**Table 4:** Thermal properties of dam environmental system.

Parameter	Unit	Proposed range	Initial value	Final value
Thermal conductivity coefficient of concrete	W/m °K	(1, 4)	1	1.7
Specific heat coefficient of concrete	W/hr·Kg °C	(0.1, 0.4)	0.1	0.25
Solar absorptivity coefficient of concrete	—	(0.6, 0.85)	0.6	0.7
Emissivity coefficient of concrete surface	—	(0.3, 0.9)	0.5	0.8
Diffuse reflectance coefficient (upstream face)	—	Table 2	0.3	0.3
Diffuse reflectance coefficient (downstream face)	—	Table 2	0.2	0.2
Convection coefficient of air	W/m^2^ °K	(5, 25)	60*	60*
Convection coefficient of water	W/m^2^ °K	Variable	6000	6000

*Considering the wind and radiation of air convection effects leads to increasing of this parameter significantly; refer to (3).
